# The Principles of Ligand Specificity on beta-2-adrenergic receptor

**DOI:** 10.1038/srep34736

**Published:** 2016-10-05

**Authors:** H. C. Stephen Chan, Slawomir Filipek, Shuguang Yuan

**Affiliations:** 1 Faculty of Life Sciences, University of Bradford, Bradford, West Yorkshire, BD7 1DP, United Kingdom; 2 Laboratory of Biomodeling, Faculty of Chemistry & Biological and Chemical Research Centre, University of Warsaw, ul. Pasteura 1, Warsaw 02-093, Poland; 3 Laboratory of Physical Chemistry of Polymers and Membranes, Ecole Polytechnique Fédérale de Lausanne (EPFL), CH B3 495 (Bâtiment CH) Station 6, Lausanne CH-1015, Switzerland

## Abstract

G protein-coupled receptors are recognized as one of the largest families of membrane proteins. Despite sharing a characteristic seven-transmembrane topology, G protein-coupled receptors regulate a wide range of cellular signaling pathways in response to various physical and chemical stimuli, and prevail as an important target for drug discovery. Notably, the recent progress in crystallographic methods led to a breakthrough in elucidating the structures of membrane proteins. The structures of β_2_-adrenergic receptor bound with a variety of ligands provide atomic details of the binding modes of agonists, antagonists and inverse agonists. In this study, we selected four representative molecules from each functional class of ligands and investigated their impacts on β_2_-adrenergic receptor through a total of 12 × 100 ns molecular dynamics simulations. From the obtained trajectories, we generated molecular fingerprints exemplifying propensities of protein-ligand interactions. For each functional class of compounds, we characterized and compared the fluctuation of the protein backbone, the volumes in the intracellular pockets, the water densities in the receptors, the domain interaction networks as well as the movements of transmembrane helices. We discovered that each class of ligands exhibits a distinct mode of interactions with mainly TM5 and TM6, altering the shape and eventually the state of the receptor. Our findings provide insightful prospective into GPCR targeted structure-based drug discoveries.

G protein-coupled receptors (GPCRs) are involved in a wide spectrum of physiological functions and they are the most attractive target for modern drug discovery. With the advances in both static crystal structures and molecular dynamics (MD) simulations, some common features in the activation mechanism of GPCRs have been identified, including the side-chain switches, the movement of transmembrane (TM) helices, and the formation of internal water channel^1^,^2^,^3^,^4^. However, an important question why certain molecules act as agonists whereas others, even with nearly identical structure, act as antagonists or invert agonists, is not well understood.

To answer this question and facilitate GPCR targeted drug discovery, we focus on the molecular fingerprint of the β_2_-adrenergic receptor (β_2_AR). β-adrenoceptors belong to rhodopsin-like GPCRs and are subdivided into three distinct groups: β_1_, β_2_, and β_3_ which are identified in cardiac, airway smooth muscle, and adipose tissue, respectively^5^. β_2_AR plays an important role in many physiological processes including inhibiting labor, delaying need of micturition, facilitating respiration and providing glucose fuel^6^,^7^. Similar to other receptors, there are three different ligand types of β_2_AR: agonists, antagonists and inverse agonists. The agonists activate the signaling pathways and increase the receptor’s basal activities. The antagonists block the signaling transductions without affecting the receptor’s basal activities. However, the inverse agonists block the receptor’s pocket and reduce the receptor’s basal activities^4^,^8^,^9^. All these three types of compounds have been extensively characterized even before their structures were determined^10^,^11^,^12^.

With the available crystal structures^9^,^13^,^14^,^15^, we are now able to envisage both active and inactive states of the receptor. Here, we further investigated the detailed binding modes of twelve different molecules (four agonists, four antagonists, and four inverse agonists) in the corresponding states of β_2_AR through 12 × 100 ns molecular dynamics (MD) simulations (a total of 1200 ns trajectories). The propensities of residue-ligand interactions were presented as interaction fingerprints. Moreover, the fluctuation of the protein backbone, the volumes in the intracellular pockets, the water densities in the receptors, the domain interaction networks as well as the movements of transmembrane helices are characterized for each class of compounds.

## Results and Discussion

### Molecular scaffolds of the agonists, antagonists and inverse agonists

Ligands are functionally categorized into three classes, namely agonists, antagonists and inverse agonists, based on their pharmacological functions. Four typical molecules were selected from each class of compounds and their protein-bound complexes were obtained from the reported crystal structures, or by protein-ligand docking if the experimental data were unavailable (Table 1). Specifically, for the agonist-bound systems, three of them were based on the crystal structures of BI167107 (Agon-1), HBI (Agon-2) and adrenaline (Agon-3) (PDB codes: 4LDE, 4LDL and 4LDO respectively)^16^, whilst the fourth one was built by docking salbutamol (Agon-4), a potent β_2_ selective agonist^17^,^18^, into the orthosteric site of an activated receptor (pdb: 4LDE). The five top ranked docking poses are identical to the agonist in the crystal structures. (Figure Suppl. 1) For the antagonist-bound systems, only the crystal structure of alprenolol (Anta-1) bound complex is available (PDB code: 3NYA)^15^. The most active enantiomer of bupranolol (Anta-2)^19^, nadolol (Anta-3)^20^ and propranolol (Anta-4)^19^ were selected for their specificity on β_2_ over β_1_ receptors^21^ and docked into the orthosteric site of an inactive receptor (pdb: 3NYA). For all four inverse agonists, their protein-bound structures are available (PDB codes: 2RH1^13^, 3D4S^22^, 3NY8^15^ and 3NY9^15^ respectively).

**Figure 1:**
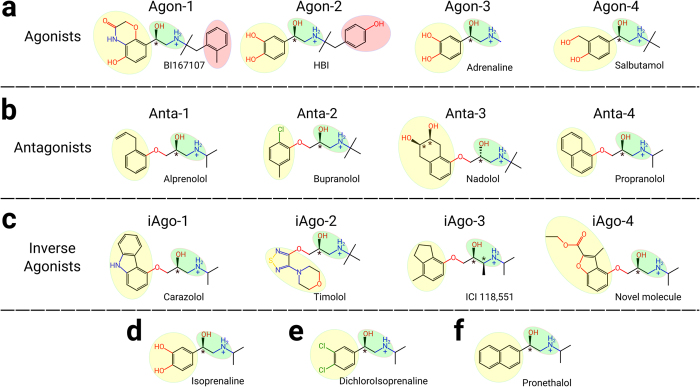
The molecular schemes of the ligands in the twelve studied systems and the important ligands during the development of beta-blockers. (**a**) The four agonists. (**b**) The four antagonists. (**c**) The four inverse agonists. (**d**) Isoprenaline (agonist). (**e**) Dichloroisoprenaline (partial agonist). (**f**) Pronethalol (antagonist). Yellow circle: ring (I); Green circle: ethanolamine, Red circle: ring II.

There are common features between each class of compounds (Fig. 1). In particular, all agonist molecules consist of an aromatic ring (ring I) connected to an ethanolamine backbone (Fig. 1a). An extra N-substituted isobutylphenyl ring (ring II) is found in both Agon-1 and Agon-2. This feature is not observed in any other ligands. Interestingly, both antagonists (Fig. 1b) and inverse agonists (Fig. 1c) contain an additional oxymethylene bridge between ring I and the ethanolamine backbone. These rings I are in general less polar and contain less Hbond donors.

### Protein ligand interaction fingerprint

**Figure 2:**
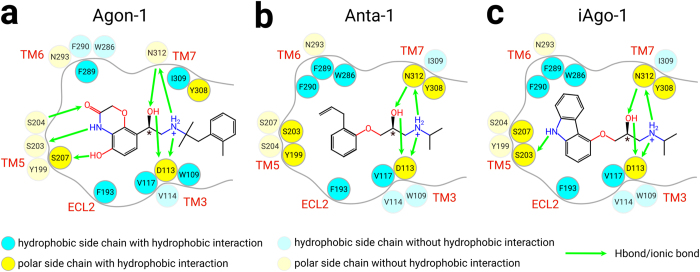
The 16 important residues that form polar and/or hydrophobic interactions with a ligand. (**a**) BI167107 (Agon-1). (**b**) Alprenolol (Anta-1). (**c**) Carazolol (iAgo-1).

**Figure 3:**
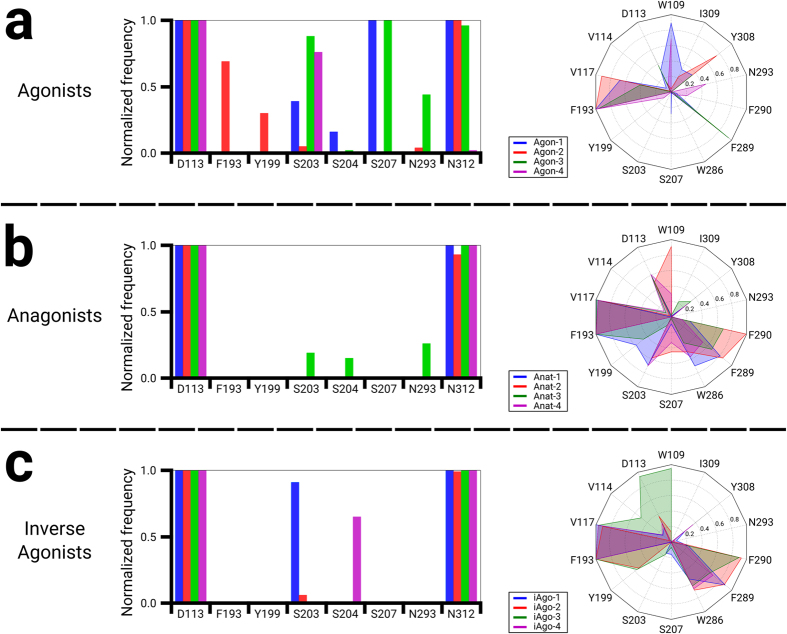
The interaction fingerprints of the twelve ligands with β2AR. (**a**) The four agonists. (**b**) The four antagonists. (**c**) The four inverse agonists. (left panel) The normalized frequency of Hbonds/ionic bonds in the final 100 frames. (right panel) The normalized frequency of hydrophobic interactions in the final 100 frames.

It is of great interest to investigate how the differences in the ligand scaffold and the receptor state affect the protein-ligand interactions. To illustrate how each ligand interacts with β_2_AR, we generated the interaction fingerprints based on the final 100 frames of our MD simulations. The root-mean-square deviations (RMSDs) of the receptor helices to the starting frame confirm the convergence of the simulations (Figure Suppl. 2). The ligands collectively interact with W109^3.28^, D113^3.32^, V114^3.33^, V117^3.36^, F193^ECL2^, Y199^5.38^, S203^5.42^, S204^5.43^, S207^5.46^, W286^6.48^, F289^6.51^, F290^6.52^, N293^6.55^, Y308^7.35^, I309^7.36^ and N312^7.39^ (Fig. 2). Noticeably, an ionic interaction is always observed between the ethanolamine of each ligand and D113^3.32^. The Hbonds between ethanolamine and N312^7.39^ are also frequently observed (frequency > 90%) in all ligands, except for Agon-4 (Fig. 3, left panel). These findings suggest that D113^3.32^ and N312^7.39^ are the key residues for the binding of all ligands. Moreover, agonists frequently form Hbonds with multiple residues in TM5, particularly with S203^5.42^ and S207^5.46^ (Fig. 3a). Interestingly, the catechol ring I of Agon-2 interacts less frequently with Y199^5.38^ and S203^5.42^ (frequency < 30%). However, its N-substituted *p*-isobutylphenol in ring II forms a prominent Hbond with the amide backbone of F193^ECL2^ (Fig. 3a, left panel). In contrast, antagonists and inverse agonists tend to have less polar interactions with TM5. Only Anta-3 can form rare Hbonds (frequency < 20%) with both S203^5.42^ and S204^5.43^ (Fig. 3b, left panel). For the inverse agonists, only iAgo-1 and iAgo-4 form isolated Hbonds with S203^5.42^ and S204^5.43^ respectively (Fig. 3c, left panel).

Apart from polar interactions, hydrophobic interactions are essential for stabilizing or activating the receptor. Rings I of all ligands are enveloped between the hydrophobic side chains of V117^3.36^, F193^ECL2^ and F289^6.51^, except that Agon-4 interacts with F193^ECL2^ and N293^6.55^ instead (Fig. 3, right panel). Moreover, we noticed that the antagonists and the inverse agonists frequently form hydrophobic interactions with Y199^5.38^, S203^5.42^, S207^5.46^, W286^6.48^ and F290^6.52^, whereas the agonists rarely form such interactions with these residues (Fig. 3b,c, right panel). The analysis of interaction fingerprint has so far indicated that polar interactions between the ligand and the residues on TM5 are necessary for maintaining the active state of the receptor, whereas a ligand can stabilize the receptor by exerting hydrophobic interactions on this helix and also TM6. Hence, the distinct interaction patterns with different regions of the receptor can help predicting the nature of such a ligand. Historically, in contrast to isoprenaline (Fig. 1d) which is a potent agonist on β-adrenergic receptor, dichloroisoprenaline (DCI) (Fig. 1e) unexpectedly demonstrated antagonistic effects on the receptor^23^,^24^. Medicinal chemists were then intrigued by the possibility of developing an antagonist via appropriate chemical modifications of these molecules. Eventually, pronethalol (Fig. 1f), the naphthyl analog of isoprenaline, was the first beta blocker as a clinical candidate. The subsequent introduction of an oxymethylene bridge between the naphthalene and ethanolamine led to the discovery of propranolol (Anta-4) (Fig. 1b), the first clinically useful beta blocker^23^,^24^. Our results are consistent with the discovery process of beta blockers that the replacement of catechol ring with nathphalene reduces the Hbond interactions on TM5 and promotes the hydrophobic interactions. Meanwhile, the additional oxymethylene facilitates the hydrophobic interactions between the N-substituted group of the ligand and the residues on TM6. Therefore, one would expect the fingerprint of isoprenaline demonstrates frequent hydrogen bonds with TM5 and rare hydrophobic interactions with TM6. On the other hand, the fingerprints of DCI and pronathalol should reveal dominant hydrophobic interactions with both TM5 and TM6. We notice that the potent agonist Agon-4 does not share the common polar (N312^7.39^) and hydrophobic interactions (V117^3.36^ and F289^6.51^) as observed in other systems (Fig. 3a, right panel). Moreover, our MD trajectory shows that Agon-4 adopts a fairly different docking pose (Figure Suppl. 3). Unlike other ligands, the ring I of Agon-4 does not penetrate deeply into the pocket and therefore no hydrophobic interactions with V117^3.36^ and F289^6.51^ are formed. Instead, the 2-methylhydroxyl group (ring I) forms a Hbond with S203^5.42^ and consequently ring I is firmly stabilized next to the extracellular loop 2 (ECL2), in the vicinity of F193^ECL2^ and N293^6.51^. Interestingly, the phenol (ring I) frequently forms an intramolecular Hbond with the 2-methylhydroxyl group (ring I). On the other hand, the N-substituted isobutyl group of Agon-4 discourages N312^7.39^ from approaching the ethanolamine backbone and thus disrupts the ligand-N312^7.39^ Hbond.

### Flexibility of each TM region

**Figure 4:**
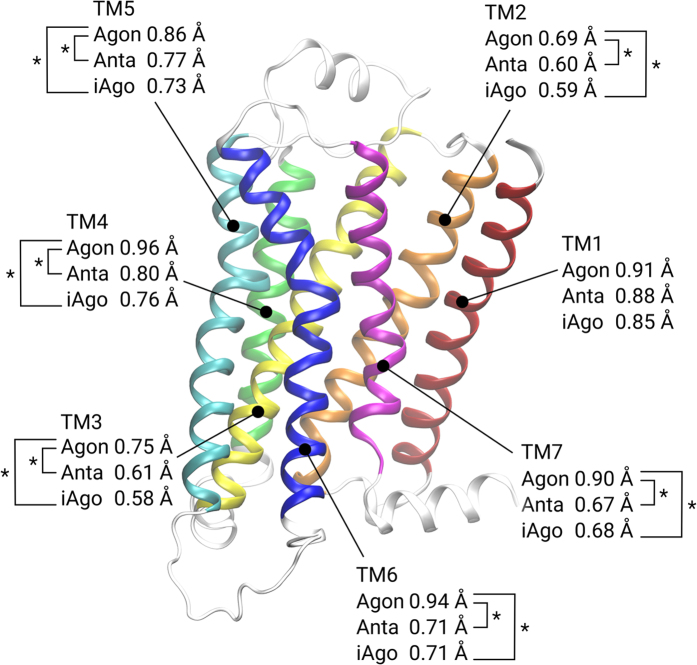
The average root-mean-square-fluctuations (RMSFs) of the protein backbone in each helix, in the presence of agonists (Agon), antagonists (Anta) or inverse agonists (iAgo). (red) TM1: W32^1.31^ to K60^1.59^. (orange) TM2: V67^2.38^ to M96^2.67^. (yellow) TM3: N103^3.22^ to T136^3.55^. (green) TM4: A150^4.42^ to M171^4.63^. (cyan) TM5: Q197^5.36^ to K227^5.66^. (blue) TM6: H269^6.31^ to I298^6.60^. (magenta) TM7: K305^7.32^ to R328^7.55^. An asterisk indicates a significant difference between the two values at *p* < 0.01, using a two-tailed t-test with equal variances.

After identifying the 16 relevant residues for ligand binding, the effect of a ligand can be reflected from the overall root-mean-square fluctuations (RMSFs) of the backbone atoms. In the presence of agonists, the average RMSF (0.69 Å) is larger than that of antagonist-bound or inverse agonist-bound systems (both 0.56 Å) and such differences are found to be statistically significant (*p* < 0.01) (Table Suppl. 1). This phenomenon is related to the fact that several Hbonds between an agonist and TM5 probably disrupt the interactions between the helices and consequently increase the protein flexibility in the transmembrane region. To investigate the global effect of an incoming ligand, the RMSF of each TM helix (defined as W32^1.31^-K60^1.59^, V67^2.38^-M96^2.67^, N103^3.22^-T136^3.55^, A150^4.42^-M171^4.63^, Q197^5.36^-K227^5.66^, H269^6.31^-I298^6.60^ and K305^7.32^-R328^7.55^ respectively) is also calculated for all ligand-bound systems. In each corresponding region, the average RMSF of agonist-bound systems (0.91, 0.69, 0.75, 0.96, 0.86, 0.94 and 0.90 Å) is always greater than that of antagonist-bound (0.88, 0.60, 0.61, 0.80, 0.77, 0.71 and 0.67 Å) and inverse agonist-bound systems (0.85, 0.59, 0.58, 0.76, 0.73, 0.71 and 0.68 Å). The RMSF differences of all corresponding helices except for TM1 are also statistically significant (*p* < 0.01) (Fig. 4). It appears that agonist molecules not only increase the flexibility near the binding site, but also the major part of the receptor.

### The intracellular pockets and water influx

**Figure 5:**
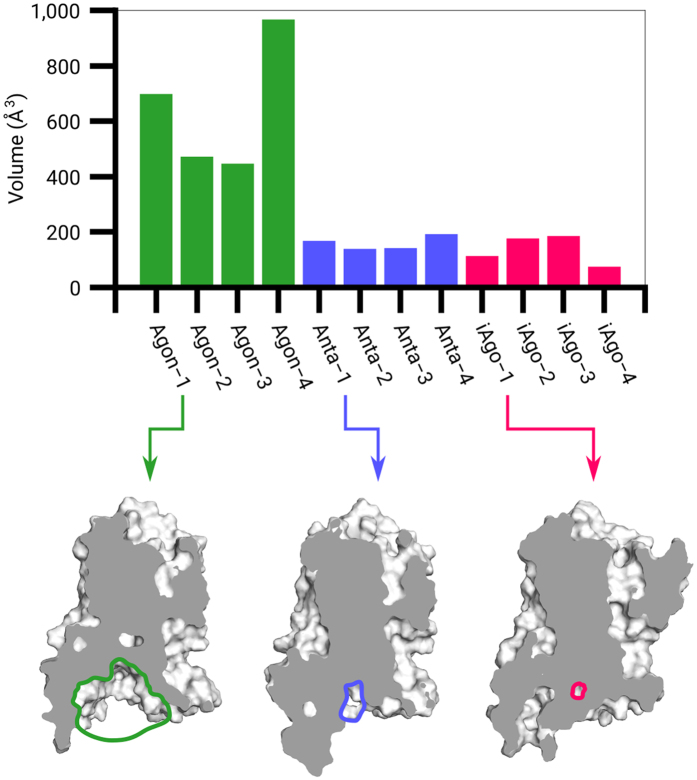
The volumes of the intracellular pockets in the twelve studied systems and the cross sections of Agon-1 bound β_2_AR, Anta-1 bound β_2_AR and iAgo-1 bound β_2_AR. (Grey) Cross section plane. (Colored circle) Intracellular pocket.

**Figure 6:**
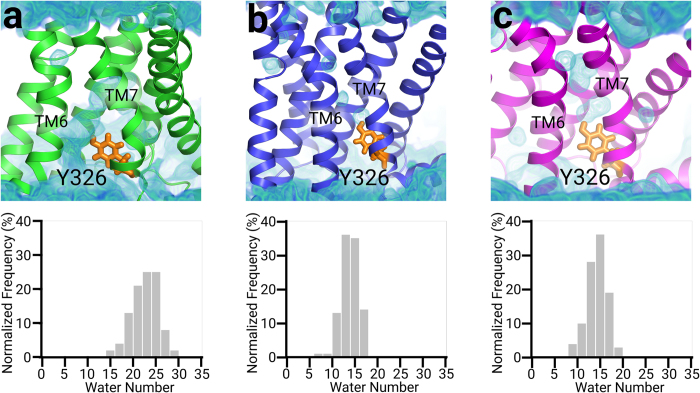
The number of water molecules next to Y326^7.53^. (**a**) Top: water density of Agon-1 bound β_2_AR in the final 100 frames. Bottom: The number of water molecules within 4 Å of Y326^7.53^ in Agon-1 bound β_2_AR. (**b**) Top: water density of Anta-1 bound β_2_AR in the final 100 frames. Bottom: The number of water molecules within 4 Å of Y326^7.53^ in Anta-1 bound β_2_AR. (**a**) Top: water density of iAgo-1 bound β_2_AR in the final 100 frames. Bottom: The number of water molecules within 4 Å of Y326^7.53^ in iAgo-1 bound β_2_AR.

Another important feature of receptor activation is the expansion of the intracellular pocket for the G protein coupling process. In this study, the last 100 frames in each trajectory were selected for calculating the average size of the intracellular pocket. For agonist-bound systems, the receptors are in their activated states and the average pocket volumes vary between ~450 and ~950 Å^3^. In contrast, the receptor remains in an inactive state in the presence of an antagonist or an inverse agonist and therefore the average pocket volumes only fluctuate between ~100 and ~200 Å^3^ (Fig. 5). Moreover, the rotamer of the highly conserved Y326^7.53^ correlates with various states of the receptor and regulates the formation of internal water channel^1^. Hence, we calculated the number of water molecules within 4 Å around Y326^7.53^ alongside the pocket volume. In Agon-1 bound β_2_AR, water influx from the cytoplasmic region into the receptor is observed and the average number of water molecules around Y326^7.53^ is 23 (Fig. 6a). In Anta-1 bound β_2_AR and iAgo-1 bound β_2_AR, there are isolated water patches that are disconnected with the bulk water in the cytoplasm. The average number of water molecules for both cases is around 14, less than two-thirds of that in the activated state (Fig. 6b,c). The details of other systems are provided in the Supplementary Information (Figure Suppl. 4).

### Domain interaction network

**Figure 7:**
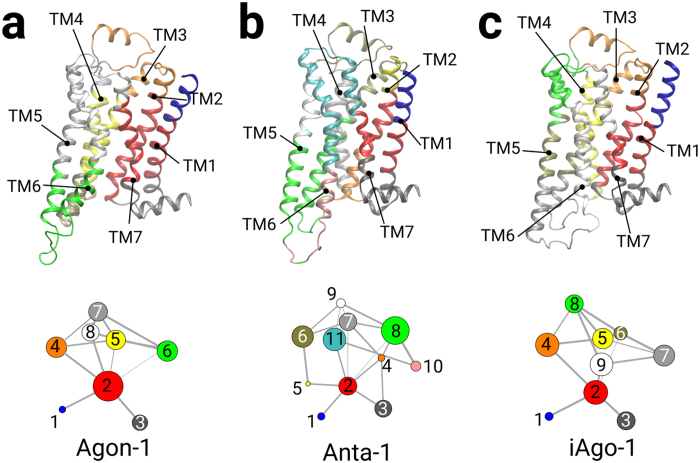
Simultaneous view of community residue interaction network and 3D structure of β_2_AR. (**a**) Agon-1 bound β_2_AR (**b**) of Anta-1 bound β_2_AR (**c**) iAgo-1 bound β_2_AR.

The activation of receptors can lead to a rearrangement of local interactions in the intracellular region^25^. In order to unravel couplings among residues within the receptor, we constructed the domain interaction networks to facilitate the analysis of state-specific couplings. Each node presents a cluster of residues in close interaction, while the thickness of the line connecting the nodes is weighted by the correlation values between the two clusters. We noticed that agonist-bound systems form less domains than the antagonist- or inverse agonist-bound systems. For example, Agon-1 has only 8 nodes whereas Anta-1 and iAgo-1 have 11 and 9 nodes respectively (Fig. 7, bottom panel). This finding suggests that there are more discrete local interactions in the inactive state of the receptor. Noticeably the position of TM6 in the intracellular region varies with the state of the receptor, leading to a different interaction network. Among all agonist-bound systems, TM6 of the activated receptor moves outwards and eventually shares the same interaction domain with TM5 (Fig. 7a, top panel). In the antagonist- and inverse agonist-bound systems, TM6 of the inactive receptor closes up the intracellular pocket and therefore the residues in TM5 cluster with those in TM3 instead (Fig. 7b,c, top panel). These findings are in agreement with our recent work on P2Y1 receptor^26^.

### Principal component analysis on the vibrational modes

**Figure 8:**
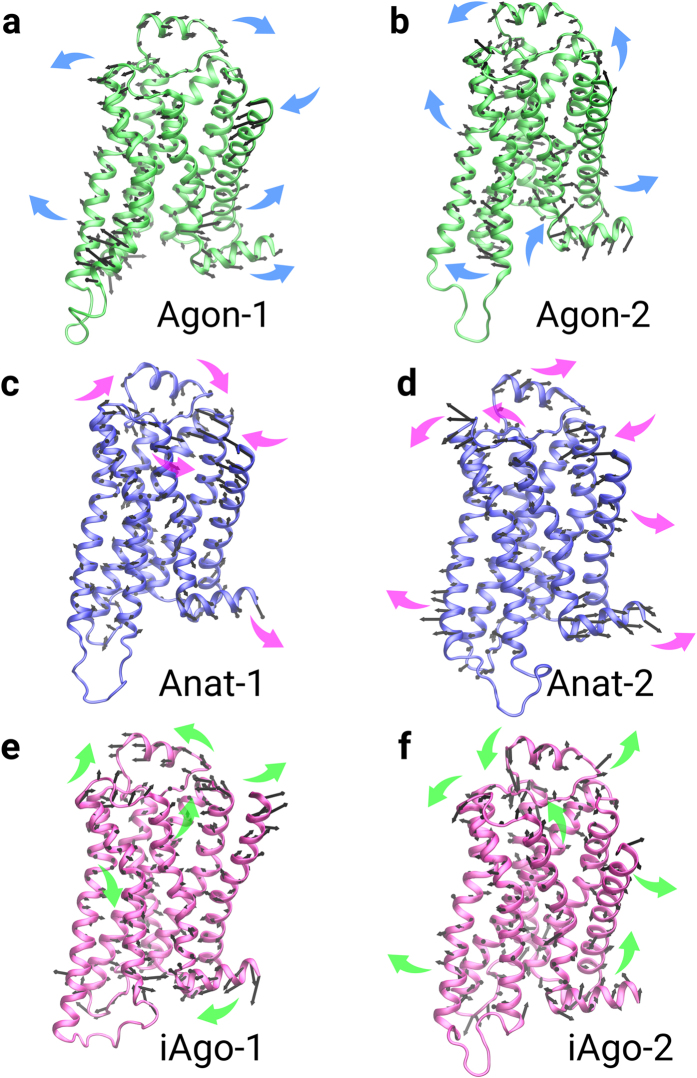
The helix movements of a ligand-bound β_2_AR. (**a**) Agon-1 bound β_2_AR. (**b**) Agon-2 bound β_2_AR. (**c**) Anta-1 bound β_2_AR. (**d**) Anta-2 bound β_2_AR. (**e**) iAgo-1 bound β_2_AR. (**f**) iAgo-2 bound β_2_AR.

The helix movements of agonist-, antagonist- and inverse agonist-bound receptors are very different in the intracellular region, but do not demonstrate consistent patterns in the extracellular region. Figure 8 shows the first and the lowest frequency mode of the alpha carbons. The proportion of variance of atom fluctuations versus eigenvalue rank is given in Figure Suppl. 5. In the extracellular region of Agon-1 bound β_2_AR, the extracellular loop 2 moves towards TM2, whereas TM5, TM6, together with extracellular loop 3 move away from TM2 and open up the ligand binding pocket (Fig. 8a). However, in Agon-2 bound β_2_AR, TM5, TM6 and the extracellular loops 2 and 3 close up the binding pocket (Fig. 8b). Similarly, in Anta-1 bound β_2_AR, TM6, TM7 and the extracellular loop 2 move towards TM2 (Fig. 8c), whereas, in Anta-2 bound β_2_AR, these groups move away from TM2 (Fig. 8d). For the iAgo-1 bound β_2_AR, the extracellular loop 2 move aside and the binding site is closed up by TM6, TM7 and the extracellular loop 3 (Fig. 8e). In contrast, the binding pocket of iAgo-2 bound β_2_AR is closed by the extracellular loop 2 (Fig. 8f). The helix movements in the intracellular region are more consistent. In agonist bound systems, TM6 and TM7 move away from each other and thus a large void space is created (Fig. 8a,b). In antagonist and inverse agonist bound systems, the movement of the alpha carbons in TM6 are more diverse and consequently the helix keeps the intracellular pocket closed (Fig. 8c–f).

## Conclusion

**Figure 9:**
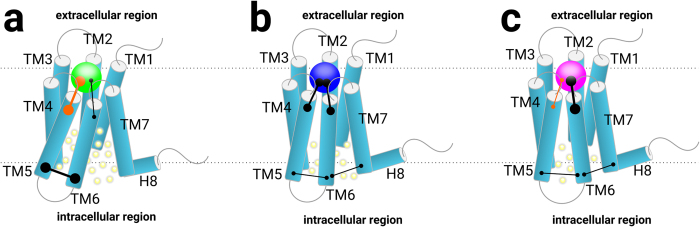
The relationship of protein-ligand interactions and the state of β_2_AR. Capped lines: (thick) dominant interactions; (thin) rare interactions; (orange) hydrogen bond interactions; (black) non-polar interactions. Yellow spheres: water molecules. (**a**) green sphere: agonist. (**b**) blue sphere: antagonist. (**c**) pink sphere: inverse agonist.

The crystal structures of β_2_AR provide new information of this classical GPCR drug target. Protein-ligand interaction fingerprints generated from MD trajectories help identify the important residues and the type of interactions required for designing ligands with desired property. In this study, using interaction fingerprints, we analyzed dynamic behavior of 16 important residues in the binding pockets, among which D113^3.32^ and N312^7.39^ are essential for ligand binding. The polar interactions with residues in TM5, particularly S203^5.42^ and S207^5.46^, are related to the agonistic properties, whereas hydrophobic interactions with residues in TM5 and TM6 help stabilize the receptor. We demonstrate that the molecular fingerprints can be a powerful tool in capturing the specific profile of protein-ligand interactions, and can be employed together with MD simulations in predicting the nature of a ligand. Agonists predominantly form Hbonds with TM5, disrupt the interactions between helices in the extracellular region and then in the rest of TM region leading to increase the flexibility of the protein. As a result, TM5 as well as TM6 form frequent non-polar interactions in the intracellular region and move away from TM7, causing the expansion of intracellular pocket and a water influx (Fig. 9a). This also explains why the residues of TM5 and TM6 in this region share the same interaction domain. In contrast, antagonists form prominently non-polar interactions with both TM5 and TM6 (Fig. 9b), whereas inverse agonists mainly form non-polar interactions with TM6 only (Fig. 9c). These modes of interactions stabilize the extracellular region of the receptor, resulting in a smaller intracellular pocket and a lower amount of water. Residues of TM5 and TM6 in the intracellular region form discrete domains and their vibrational modes become more diverse. All these findings explain the fundamental mechanism of receptor activation and use as good guidance for GPCR targeted drug discoveries.

## Methods

### Loop filling and refinements

All fusion proteins, lipids, counterions and stabilizing agents were removed from the experimental structures. The missing intracellular loop ICL2 in each receptor was rebuilt and refined using the loop refinement protocol in Modeller^27^ V9.10. A total of 2,000 loops were generated for each receptor and the conformation with the lowest DOPE (Discrete Optimized Protein Energy) score was chosen for receptor construction. Repaired models were submitted to Rosetta V3.4 for loop refinement with kinematic loop modeling methods^28^. Kinematic closure (KIC) is an analytic calculation inspired by robotics techniques for rapidly determining the possible conformations of linked objects subject to constraints. In the Rosetta KIC implementation, 2N - 6 backbone torsions of an N-residue peptide segment (called non-pivot torsions) are set to values drawn randomly from the Ramachandran space of each residue type, and the remaining 6 phi/psi torsions (called pivot torsions) are solved analytically by KIC.

### Protein structure preparations

All protein models were prepared in Schrodinger suite software under OPLS_2005 force field^29^. Hydrogen atoms were added to repaired crystal structures according to the physiological pH (7.0) with the PROPKA^30^ tool in Protein Preparation tool in Maestro^31^ (2015, Schrödinger 2015) to optimize the hydrogen bond network. Constrained energy minimizations were conducted on the full-atomic models, with heavy atom coverage to 0.4 Å.

### Ligand structure preparations and docking

All ligand structures were obtained from the PubChem^32^ online database. The LigPrep module in Schrodinger 2015 suite software was introduced for geometric optimization by using OPLS_2005 force field. The ionization state of ligands were calculated with Epik^33^ tool employing Hammett and Taft methods in conjunction with ionization and tautomerization tools^33^. For salbutamol (Agon-1), bupranolol (Anta-2), nadolol (Anta-3) and propranolol (Anta-4), flexible ligand docking calculations were performed using Glide module^34^. These molecules were docked into the ligand binding site of 4LDE for the agonist and that of 3NYA for the antagonists. The best pose with the lowest GScore was selected for MD simulations.

### Molecular dynamics simulations

The membrane system was built in Desmond^35^, with the receptor crystal structure pre-aligned in the OPM (Orientations of Proteins in Membranes) database^36^,^37^. Pre-equilibrated 76-92 POPC lipids coupled with 6900-9200 TIP3P water molecules in a box ~ 65 Å x 63 Å x 93 Å were used for building the protein/membrane system. We modeled the protein, lipids, water and ions by using CHARMM36 force field^38^. The ligand was assigned with CHARMM CGenFF force field^39^. Next, an additional 20 ns constrained equilibration was performed at constant pressure and temperature (NPT ensemble; 310 K, 1 bar), and the force constant was trapped off gradually from 10 kcal/mol to 0 kcal/mol. All bond lengths to hydrogen atoms were constrained with M-SHAKE. Van der Waals and short-range electrostatic interactions were cut off at 10 Å. Long-range electrostatic interactions were computed by the Particle Mesh Ewald (PME) summation scheme. All MD simulations were done in Desmond^35^ with 40 ps step size. The root-mean-square fluctuations (RMSFs) of the protein backbone were calculated in Gromacs^40^. The root-mean-square deviations (RMSDs) of the protein helices and the principal component analysis (PCA) of the alpha carbons in the final 50 frames were calculated in VMD^41^.

### Average water density calculation

Water density was calculated in Volmap plugin in VMD^41^. Volmap creates a map of the weighted atomic density at each grid point. This is done by replacing each atom in the selection with a normalized gaussian distribution of width (standard deviation) equal to its atomic radius. The gaussian distribution for each atom is then weighted using an optional weight read from one of the atoms’ numerical properties, and defaults to a weight of one. The various gaussians are then additively distributed on a grid. The meaning of final map will depend of the weights of mass. The average water density was calculated based on the final 100 frames of each long time scale MD simulations. Final output results were visualized in VMD.

### Interaction fingerprint calculations

The interaction fingerprint between protein and ligand was done with IChem^42^. We first extracted the final 100 snapshots of the MD simulation, and shortlisted the residues with atoms less than 5 Å away from any ligand atoms. We then used IChem to convert protein−ligand coordinates into a bit-string fingerprint (TIFP) registering the corresponding molecular interaction pattern. TIFP fingerprints have been calculated for ca. 1000 protein−ligand complexes, enabling a broad comparison of relationships between interaction pattern similarities and ligand or binding site pairwise similarities^42^. In this work we kept the default parameters of IChem and focused on two types of interactions: polar interaction and hydrophobic contact. The former comprises ionic bond and Hbond, whilst the latter incorporates hydrophobic contacts, the face-to-face and edge-to-face between aromatic rings. 16 residues formed interactions with one of the ligands for at least 30 times.

### Residue communication network calculation

Correlated atomic fluctuations of a particular receptor state were characterized as reported elsewhere^43^,^44^,^45^ using Bio3D. The network nodes represent residues, which are connected through edges weighted by their constituent atomic correlation values. Community analysis and node centrality with Bio3D and suboptimal path calculation with the WISP software^46^ were performed on each network to characterize network properties and to identify residues involved in the dynamic coupling of distal sites. The parameters for the suboptimal path analysis included input source and sink nodes, as well as the total number of paths to be calculated. The latter parameter was set to 500 paths, which was found to yield converged results in all cases^45^.

## Additional Information

**How to cite this article**: Chan, H. C. S. *et al*. The Principles of Ligand Specificity on beta-2-adrenergic receptor. *Sci. Rep.* **6**, 34736; doi: 10.1038/srep34736 (2016).

## Supplementary Material

Supplementary Information

## Figures and Tables

**Table 1:** The details of the ligands in the twelve studied systems.

Category	Label	Ligand	PDB code
Agonists	Agon-1	BI167107	4LDE
	Agon-2	HBI	4LDL
	Agon-3	Adrenaline	4LDO
	Agon-4	Salbutamol	- a
Antagonists	Anta-1	Alprenolol	3NYA
	Anta-2	Bupranolol	- a
	Anta-3	Nadolol	- a
	Anta-4	Propranolol	- a
Inverse Agonists	iAgo-1	Carazolol	2RH1
	iAgo-2	Timolol	3D4S
	iAgo-3	ICI 118,551	3NY8
	iAgo-4	Novel molecule^b^	3NY9

^a^The ligand-β_2_AR structures are not available in the PDB. The agonist molecule is docked in the binding site in 4LDE, whilst each antagonist is docked in that of 3NYA. The complex with the best Glide score was chosen for MD simulation.

^b^See Fig. 1.

## References

[b1] Yuan S., Filipek S., Palczewski K. & Vogel H. Activation of G-protein-coupled receptors correlates with the formation of a continuous internal water pathway. Nat Commun 5, 4733, 10.1038/ncomms5733 (2014). 25203160

[b2] Tehan B. G., Bortolato A., Blaney F. E., Weir M. P. & Mason J. S. Unifying family A GPCR theories of activation. Pharmacol Ther 143, 51–60, 10.1016/j.pharmthera.2014.02.004 (2014). 24561131

[b3] Trzaskowski B. . Action of molecular switches in GPCRs - theoretical and experimental studies. Curr Med Chem 19, 1090–1109 (2012). 22300046 10.2174/092986712799320556 PMC3343417

[b4] Granier S. & Kobilka B. A new era of GPCR structural and chemical biology. Nature chemical biology 8, 670–673, 10.1038/nchembio.1025 (2012). 22810761 PMC4031315

[b5] Johnson M. Molecular mechanisms of beta(2)-adrenergic receptor function, response, and regulation. The Journal of allergy and clinical immunology 117, 18–24, quiz 25, 10.1016/j.jaci.2005.11.012 (2006). 16387578

[b6] Hen R., Axel R. & Obici S. Activation of the beta 2-adrenergic receptor promotes growth and differentiation in thyroid cells. Proceedings of the National Academy of Sciences of the United States of America 86, 4785–4788 (1989). 2471981 10.1073/pnas.86.12.4785 PMC287358

[b7] von Heyden B. . Response of guinea pig smooth and striated urethral sphincter to cromakalim, prazosin, nifedipine, nitroprusside, and electrical stimulation. Neurourology and urodynamics 14, 153–168 (1995). 7540086 10.1002/nau.1930140208

[b8] Kobilka B. The structural basis of G-protein-coupled receptor signaling (Nobel Lecture). Angewandte Chemie 52, 6380–6388, 10.1002/anie.201302116 (2013). 23650120 PMC4031317

[b9] Rasmussen S. G. F. . Crystal structure of the beta(2) adrenergic receptor-Gs protein complex. Nature 477, 549–U311, Doi 10.1038/Nature10361 (2011). 21772288 PMC3184188

[b10] Cazzola M., Calzetta L. & Matera M. G. beta(2) -adrenoceptor agonists: current and future direction. British journal of pharmacology 163, 4–17, 10.1111/j.1476-5381.2011.01216.x (2011). 21232045 PMC3085864

[b11] Hillman K. L., Doze V. A. & Porter J. E. Functional characterization of the beta-adrenergic receptor subtypes expressed by CA1 pyramidal cells in the rat hippocampus. The Journal of pharmacology and experimental therapeutics 314, 561–567, 10.1124/jpet.105.084947 (2005). 15908513

[b12] Nathan R. A. . Comparison of the bronchodilator effects of nebulized bitolterol mesylate and isoproterenol hydrochloride in steroid-dependent asthma. The Journal of allergy and clinical immunology 79, 822–829 (1987). 3571773 10.1016/0091-6749(87)90216-8

[b13] Cherezov V. . High-resolution crystal structure of an engineered human beta2-adrenergic G protein-coupled receptor. Science 318, 1258–1265, 10.1126/science.1150577 (2007). 17962520 PMC2583103

[b14] Rasmussen S. G. . Crystal structure of the human beta2 adrenergic G-protein-coupled receptor. Nature 450, 383–387, 10.1038/nature06325 (2007). 17952055

[b15] Wacker D. . Conserved binding mode of human beta2 adrenergic receptor inverse agonists and antagonist revealed by X-ray crystallography. Journal of the American Chemical Society 132, 11443–11445, 10.1021/ja105108q (2010). 20669948 PMC2923663

[b16] Ring A. M. . Adrenaline-activated structure of beta2-adrenoceptor stabilized by an engineered nanobody. Nature 502, 575–579, 10.1038/nature12572 (2013). 24056936 PMC3822040

[b17] Prime F. J. Adrenergic Receptors, Bronchodilators and Asthma. Drugs 1, 269–273, 10.2165/00003495-197101040-00001 (1971). 4398802

[b18] Ind P. W. Salbutamol enantiomers: early clinical evidence in humans. Thorax 52, 839–840, 10.1136/thx.52.10.839 (1997). 9404366 PMC1758429

[b19] Martin L. J. . Differences in the Antinociceptive Effects and Binding Properties of Propranolol and Bupranolol Enantiomers. J Pain 16, 1321–1333, 10.1016/j.jpain.2015.09.004 (2015). 26456674

[b20] Wang X. & Ching C. B. Liquid chromatographic retention behavior and enantiomeric separation of three chiral center beta-blocker drug (nadolol) using heptakis (6-azido-6-deoxy-2, 3-di-O-phenylcarbamolyted) beta-cyclodextrin bonded chiral stationary phase. Chirality 14, 798–805, 10.1002/chir.10141 (2002). 12395397

[b21] Ignarro L. J. Different pharmacological properties of two enantiomers in a unique beta-blocker, nebivolol. Cardiovasc Ther 26, 115–134, 10.1111/j.1527-3466.2008.00044.x (2008). 18485134

[b22] Hanson M. A. . A specific cholesterol binding site is established by the 2.8 A structure of the human beta2-adrenergic receptor. Structure 16, 897–905, 10.1016/j.str.2008.05.001 (2008). 18547522 PMC2601552

[b23] Black J. Drugs from emasculated hormones: the principle of syntopic antagonism. Science 245, 486–493, 10.1126/science.2569237 (1989). 2569237

[b24] Quirke V. Putting theory into practice: James Black, receptor theory and the development of the beta-blockers at ICI, 1958–1978. Med Hist 50, 69–92, 10.1017/S0025727300009455 (2006). 16502872 PMC1369014

[b25] Yuan S., Palczewski K., Peng Q., Kolinski M. Vogel H. & Filipek S. The Mechanism of Ligand-Induced Activation or Inhibition of μ- and κ-Opioid Receptors. Angewandte Chemie 54, 7560–7563, 10.1002/anie.201501742 (2015). 25968837

[b26] Yuan S., Chan H. C., Vogel H., Filipek S. Stevens R. C. & Palczewski K. The Molecular Mechanism of P2Y1 Receptor Activation. Angew Chem Int Ed Engl 55, 10331–10335, 10.1002/anie.201605147 (2016). 27460867 PMC4996126

[b27] Eswar N. . Comparative protein structure modeling using MODELLER. Current protocols in protein science / editorial board, John E. Coligan … [et al.] Chapter 2, Unit 2 9, 10.1002/0471140864.ps0209s50 (2007). 18429317

[b28] Mandell D. J., Coutsias E. A. & Kortemme T. Sub-angstrom accuracy in protein loop reconstruction by robotics-inspired conformational sampling. Nature methods 6, 551–552, 10.1038/nmeth0809-551 (2009). 19644455 PMC2847683

[b29] Shivakumar D. . Prediction of Absolute Solvation Free Energies using Molecular Dynamics Free Energy Perturbation and the OPLS Force Field. J Chem Theory Comput 6, 1509–1519, 10.1021/Ct900587b (2010). 26615687

[b30] Sondergaard C. R., Olsson M. H., Rostkowski M. & Jensen J. H. Improved Treatment of Ligands and Coupling Effects in Empirical Calculation and Rationalization of pKa Values. J Chem Theory Comput 7, 2284–2295, 10.1021/ct200133y (2011). 26606496

[b31] Maestro, version 9.3.5 (Schrödinger, LLC, 2015).

[b32] Wang Y. . PubChem’s BioAssay Database. Nucleic Acids Res 40, D400–D412, 10.1093/nar/gkr1132 (2012). 22140110 PMC3245056

[b33] Greenwood J. R., Calkins D., Sullivan A. P. & Shelley J. C. Towards the comprehensive, rapid, and accurate prediction of the favorable tautomeric states of drug-like molecules in aqueous solution. Journal of computer-aided molecular design 24, 591–604, 10.1007/s10822-010-9349-1 (2010). 20354892

[b34] Lambert S. M. & Childers S. R. Modification of guanine nucleotide-regulatory components in brain membranes. I. Changes in guanosine 5′-triphosphate regulation of opiate receptor-binding sites. The Journal of neuroscience : the official journal of the Society for Neuroscience 4, 2755–2763 (1984). 6094741 10.1523/JNEUROSCI.04-11-02755.1984 PMC6564730

[b35] Chow E, Bowers R. C. Dror KJ Hughes RO & Desmond DH. performance on a cluster of multicore processors. D. E. Shaw Research Technical Report DESRES/TR 1 (2008).

[b36] Lomize A. L., Pogozheva I. D. & Mosberg H. I. Anisotropic solvent model of the lipid bilayer. 1. Parameterization of long-range electrostatics and first solvation shell effects. Journal of chemical information and modeling 51, 918–929, 10.1021/ci2000192 (2011). 21438609 PMC3089899

[b37] Lomize A. L., Pogozheva I. D. & Mosberg H. I. Anisotropic solvent model of the lipid bilayer. 2. Energetics of insertion of small molecules, peptides, and proteins in membranes. Journal of chemical information and modeling 51, 930–946, 10.1021/ci200020k (2011). 21438606 PMC3091260

[b38] Klauda J. B. . Update of the CHARMM all-atom additive force field for lipids: validation on six lipid types. J Phys Chem B 114, 7830–7843, 10.1021/jp101759q (2010). 20496934 PMC2922408

[b39] Vanommeslaeghe K., Raman E. P. & MacKerell A. D. Jr. Automation of the CHARMM General Force Field (CGenFF) II: assignment of bonded parameters and partial atomic charges. Journal of chemical information and modeling 52, 3155–3168, 10.1021/ci3003649 (2012). 23145473 PMC3528813

[b40] Pronk S. . GROMACS 4.5: a high-throughput and highly parallel open source molecular simulation toolkit. Bioinformatics 29, 845–854, 10.1093/bioinformatics/btt055 (2013). 23407358 PMC3605599

[b41] Humphrey W., Dalke A. & Schulten K. VMD: visual molecular dynamics. J Mol Graph 14 **(33–38)**, 27–38, 10.1016/0263-7855(96)00018-5 (1996). 8744570

[b42] Marcou G. & Rognan D. Optimizing fragment and scaffold docking by use of molecular interaction fingerprints. Journal of chemical information and modeling 47, 195–207, 10.1021/ci600342e (2007). 17238265

[b43] Skjaerven L., Yao X. Q., Scarabelli G. & Grant B. J. Integrating protein structural dynamics and evolutionary analysis with Bio3D. BMC bioinformatics 15, 399, 10.1186/s12859-014-0399-6 (2014). 25491031 PMC4279791

[b44] Grant B. J., Rodrigues A. P., ElSawy K. M., McCammon J. A. & Caves L. S. Bio3d: an R package for the comparative analysis of protein structures. Bioinformatics 22, 2695–2696, 10.1093/bioinformatics/btl461 (2006). 16940322

[b45] Scarabelli G. & Grant B. J. Kinesin-5 allosteric inhibitors uncouple the dynamics of nucleotide, microtubule, and neck-linker binding sites. Biophysical journal 107, 2204–2213, 10.1016/j.bpj.2014.09.019 (2014). 25418105 PMC4223232

[b46] Van Wart A. T., Durrant J., Votapka L. & Amaro R. E. Weighted Implementation of Suboptimal Paths (WISP): An Optimized Algorithm and Tool for Dynamical Network Analysis. J Chem Theory Comput 10, 511–517, 10.1021/ct4008603 (2014). 24803851 PMC3958135

