# Supplementary material for: The Principles of Ligand Specificity on beta-2-adrenergic receptor
Source: Sci Rep. 2016 Oct 5;6:34736. doi: 10.1038/srep34736 (PMC5050457; doi:10.1038/srep34736)
Supplement: Supplementary Information [file srep34736-s1.pdf]

The Principles of Ligand Specificity on beta-2-adrenergic receptor

H. C. Stephen Chan[1], Slawomir Filipek[*2] and Shuguang Yuan[*3]

[1] Faculty of Life Sciences, University of Bradford, Bradford, West Yorkshire, BD7 1DP, United Kingdom

[2] Laboratory of Biomodeling, Faculty of Chemistry & Biological and Chemical Research Centre, University of Warsaw, ul. Pasteura 1, Warsaw 02-093, Poland

[3] Laboratory of Physical Chemistry of Polymers and Membranes, Ecole Polytechnique Fédérale de Lausanne (EPFL), CH B3 495 (Bâtiment CH) Station 6, Lausanne CH-1015, Switzerland

*to whom correspondence should be addressed:

sfilipek@chem.uw.edu.pl, shuguang.yuan@gmail.com

Supporting Information

Table Suppl. 1

Figure Suppl. 1-5

Table Suppl. 1a| The average root-mean-square-fluctuations (RMSFs) of the 16 residues responsible for protein-ligand interactions, in the presence of agonists, antagonists and inverse agonists.

Residues	Backbone atoms	Average RMSFs (Å)		
		Agonists	Antagonists	Inverse agonists
$W109^{3.28}$	N	0.640	0.517	0.524
	Cα	0.599	0.488	0.490
	C	0.569	0.458	0.454
$D113^{3.32}$	N	0.493	0.407	0.405
	Cα	0.500	0.414	0.407
	C	0.504	0.421	0.416
$V114^{3.33}$	N	0.485	0.391	0.395
	Cα	0.497	0.401	0.404
	C	0.500	0.415	0.414
$V117^{3.36}$	N	0.524	0.441	0.433
	Cα	0.544	0.456	0.440
	C	0.535	0.448	0.426
$F193^{ECL2}$	N	0.819	0.692	0.655
	Cα	0.824	0.674	0.638
	C	0.800	0.674	0.640
$Y199^{5.38}$	N	0.720	0.731	0.702
	Cα	0.696	0.698	0.681
	C	0.692	0.635	0.641
$S203^{5.42}$	N	0.713	0.573	0.568
	Cα	0.719	0.568	0.552
	C	0.707	0.549	0.542
$S204^{5.43}$	N	0.698	0.540	0.539
	Cα	0.715	0.564	0.568
	C	0.735	0.578	0.578
$S207^{5.46}$	N	0.801	0.607	0.589
	Cα	0.811	0.623	0.604
	C	0.751	0.618	0.595
$W286^{6.48}$	N	0.664	0.528	0.507
	Cα	0.666	0.525	0.505
	C	0.679	0.544	0.531
$F289^{6.51}$	N	0.680	0.582	0.576
	Cα	0.642	0.553	0.554
	C	0.630	0.535	0.543
$F290^{6.52}$	N	0.644	0.537	0.544
	Cα	0.663	0.550	0.556
	C	0.728	0.588	0.599
$N293^{6.55}$	N	0.760	0.673	0.691
	Cα	0.826	0.724	0.741
	C	0.882	0.743	0.783

Table Suppl. 1a (Cont'd)

Residues	Backbone atoms	Average RMSFs (Å)		
		Agonists	Antagonists	Inverse agonists
$Y308^{7.35}$	N	0.859	0.722	0.732
	Cα	0.831	0.680	0.692
	C	0.804	0.628	0.634
$I309^{7.36}$	N	0.841	0.639	0.646
	Cα	0.848	0.632	0.638
	C	0.791	0.589	0.595
$N312^{7.39}$	N	0.626	0.504	0.512
	Cα	0.639	0.484	0.497
	C	0.625	0.482	0.495

Table Suppl. 1b| The results of two-tailed t-tests assuming equal variances at *p* < 0.01.

Data Statistics	Agon vs. Anta		Agon vs. iAgo		Anta vs. iAgo	
	Agon	Anta	Agon	iAgo	Anta	iAgo
Mean	0.685682	0.56288	0.685682	0.559682	0.56288	0.559682
Variance	0.012928	0.009618	0.012928	0.009831	0.009618	0.009831
Observations	48	48	48	48	48	48
Pooled Variance	0.011273		0.01138		0.009725	
Hypothesized Mean Difference	0		0		0	
t Stat	5.666115		5.786397		0.158865	
P(T<=t) two-tail	1.59E-07		9.41E-08		0.874116	
t Critical two-tail	2.629148		2.629148		2.629148	

* Agon, Anta and iAgo stand for agonists, antagonists, and inverse agonists.

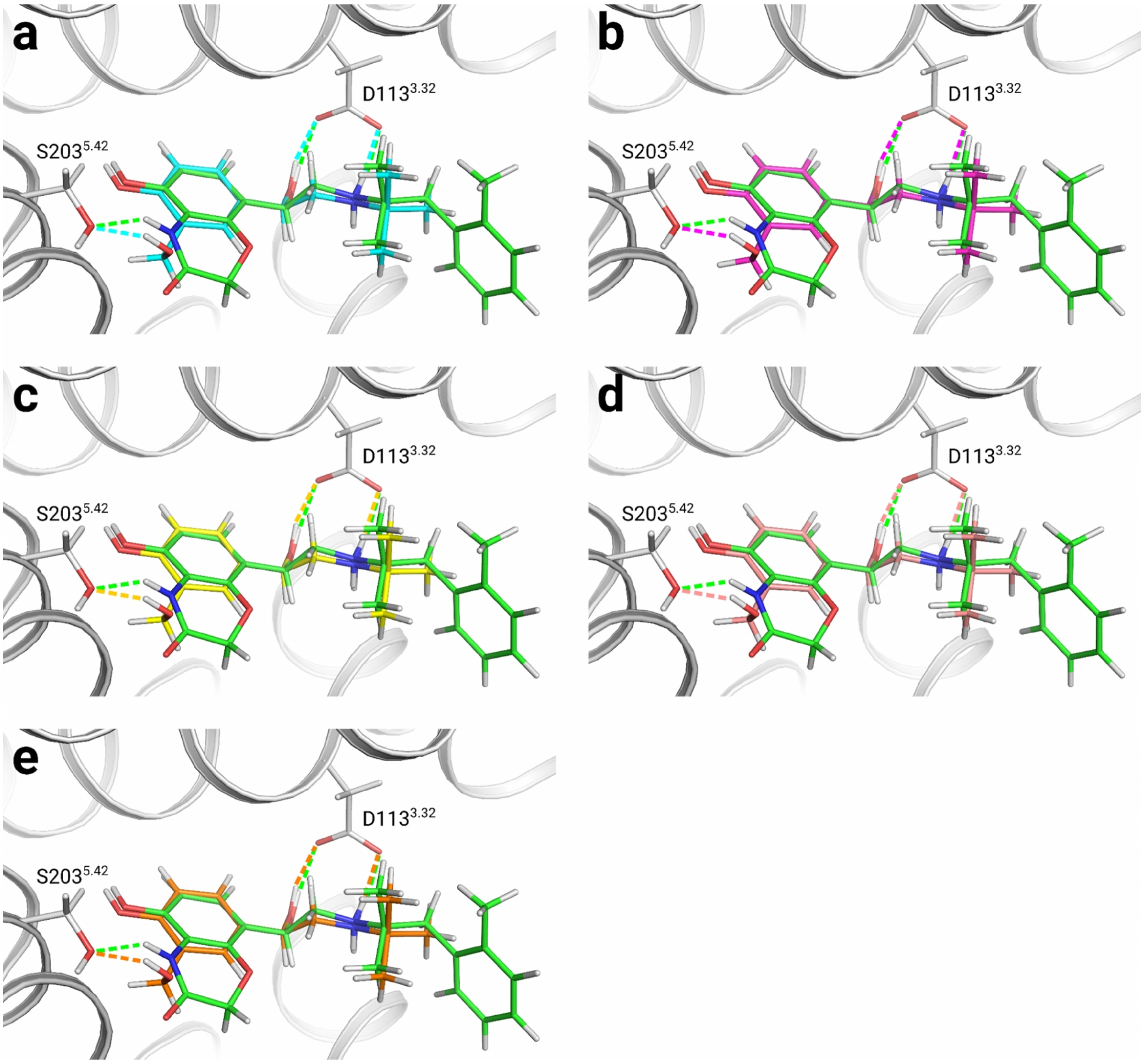

Figure Suppl. 1| The overlays of the best five docking poses of salbutamol (Agon-4) on 4LDE. (a) (green) BI167107 (Agon-1) in 4LDE; (cyan) the rank 1 pose of salbutamol (Agon-4). (b) (green) BI167107 (Agon-1) in 4LDE; (magenta) the rank 2 pose of salbutamol (Agon-4). (c) (green) BI167107 (Agon-1) in 4LDE; (yellow) the rank 3 pose of salbutamol (Agon-4). (d) (green) BI167107 (Agon-1) in 4LDE; (pink) the rank 4 pose of salbutamol (Agon-4). (e) (green) BI167107 (Agon-1) in 4LDE; (orange) the rank 5 pose of salbutamol (Agon-4).

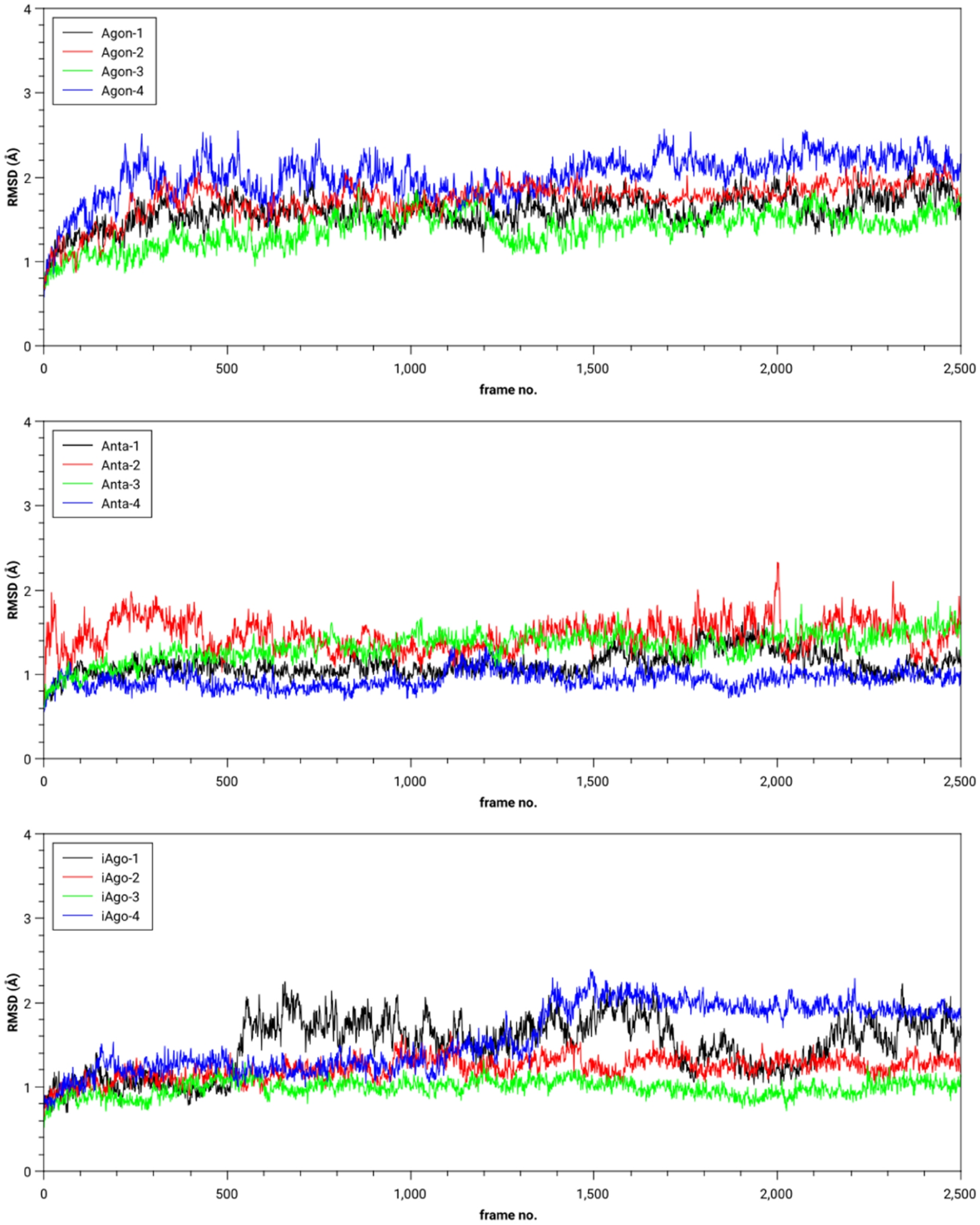

Figure Suppl. 2| The root-mean-square deviations (RMSDs) of the protein helices with respect to the starting frame.

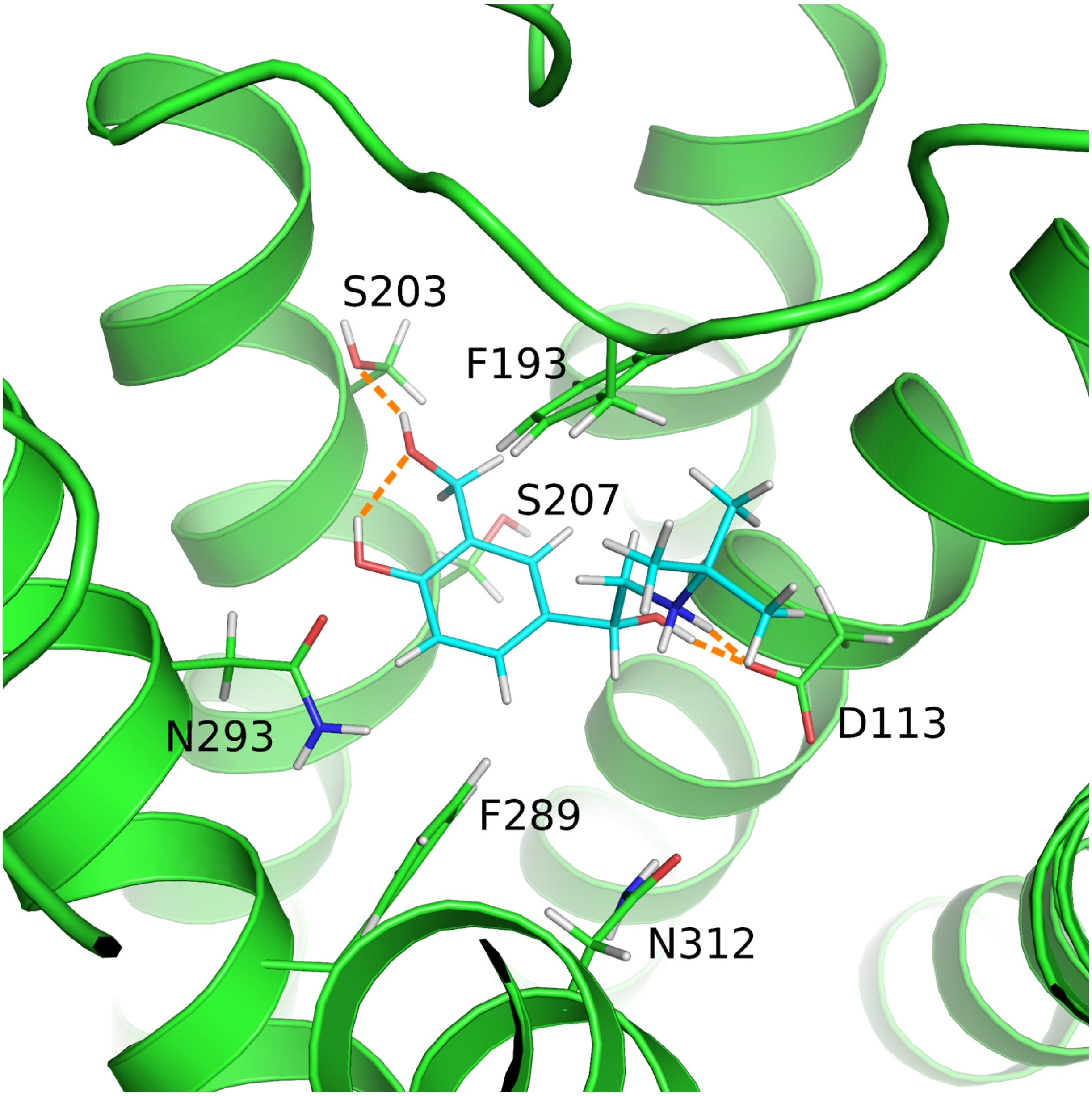

Figure Suppl. 3| The docking pose of salbutamol (Agon-4).

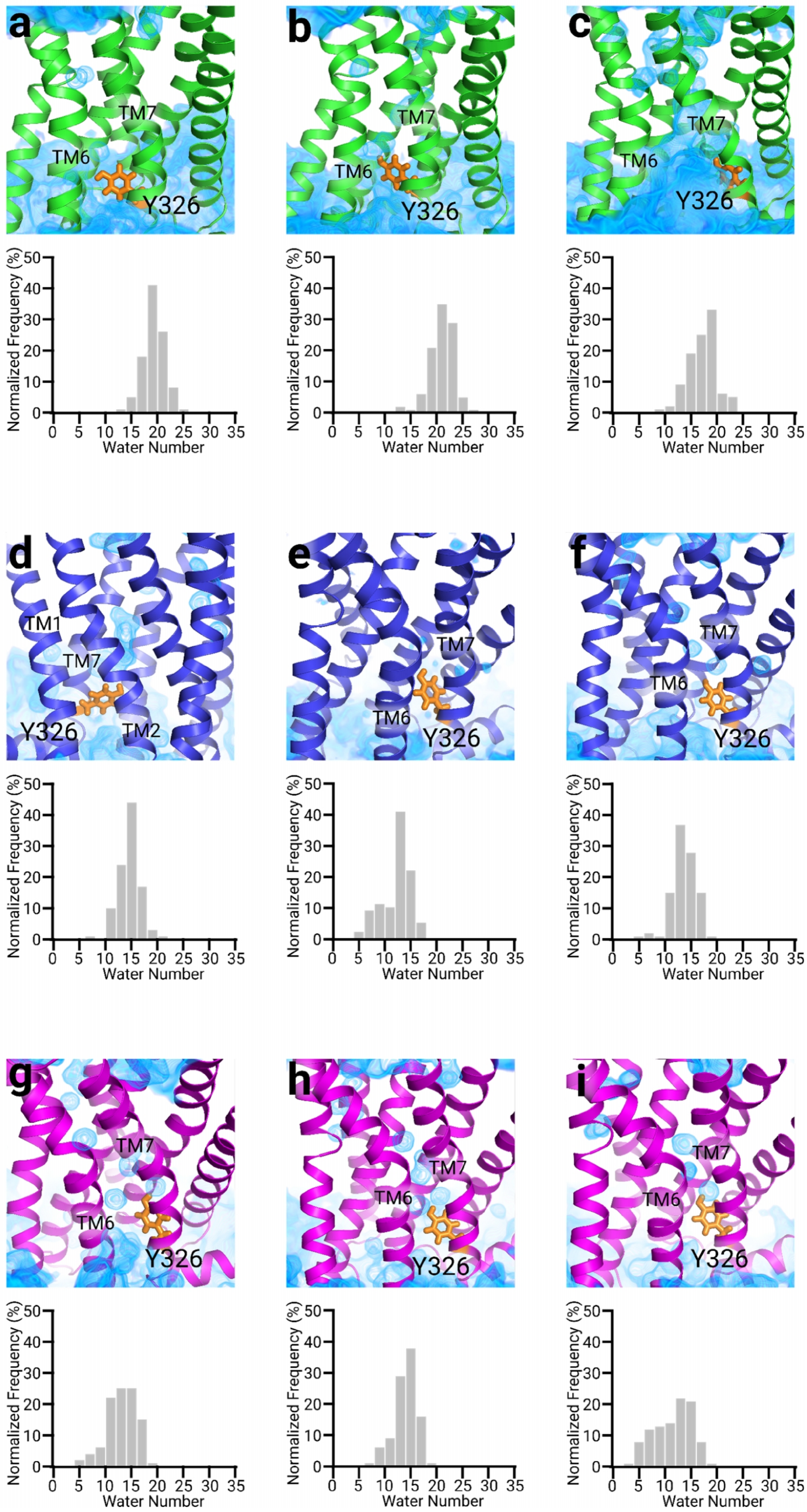
a
TM7
TM6
Y326
b
TM7
TM6
Y326
c
TM7
TM6
Y326
Normalized Frequency (%)
Water Number
d
TM1
TM7
Y326
TM2
e
TM7
TM6
Y326
f
TM7
TM6
Y326
g
TM7
TM6
Y326
h
TM7
TM6
Y326
i
TM7
TM6
Y326

Figure Suppl. 4| The number of water molecules next to Y326$^{7.53}$. (a) Top: water density of Agon-2 bound β_2AR in the final 100 frames. Bottom: The number of water molecules within 4 Å of Y326$^{7.53}$ in Agon-2 bound β_2AR. (b) Top: water density of Agon-3 bound β_2AR in the final 100 frames. Bottom: The number of water molecules within 4 Å of Y326$^{7.53}$ in Agon-3 bound β_2AR. (c) Top: water density of Agon-4 bound β_2AR in the final 100 frames. Bottom: The number of water molecules within 4 Å of Y326$^{7.53}$ in Agon-4 bound β_2AR. (d) Top: water density of Anta-2 bound β_2AR in the final 100 frames. Bottom: The number of water molecules within 4 Å of Y326$^{7.53}$ in Anat-2 bound β_2AR. (e) Top: water density of Anat-3 bound β_2AR in the final 100 frames. Bottom: The number of water molecules within 4 Å of Y326$^{7.53}$ in Anat-3 bound β_2AR. (f) Top: water density of Anat-4 bound β_2AR in the final 100 frames. Bottom: The number of water molecules within 4 Å of Y326$^{7.53}$ in Anat-4 bound β_2AR. (g) Top: water density of iAgo-2 bound β_2AR in the final 100 frames. Bottom: The number of water molecules within 4 Å of Y326$^{7.53}$ in iAgo-2 bound β_2AR. (h) Top: water density of iAgo-3 bound β_2AR in the final 100 frames. Bottom: The number of water molecules within 4 Å of Y326$^{7.53}$ in iAgo-3 bound β_2AR. (i) Top: water density of iAgo-4 bound β_2AR in the final 100 frames. Bottom: The number of water molecules within 4 Å of Y326$^{7.53}$ in iAgo-4 bound β_2AR.

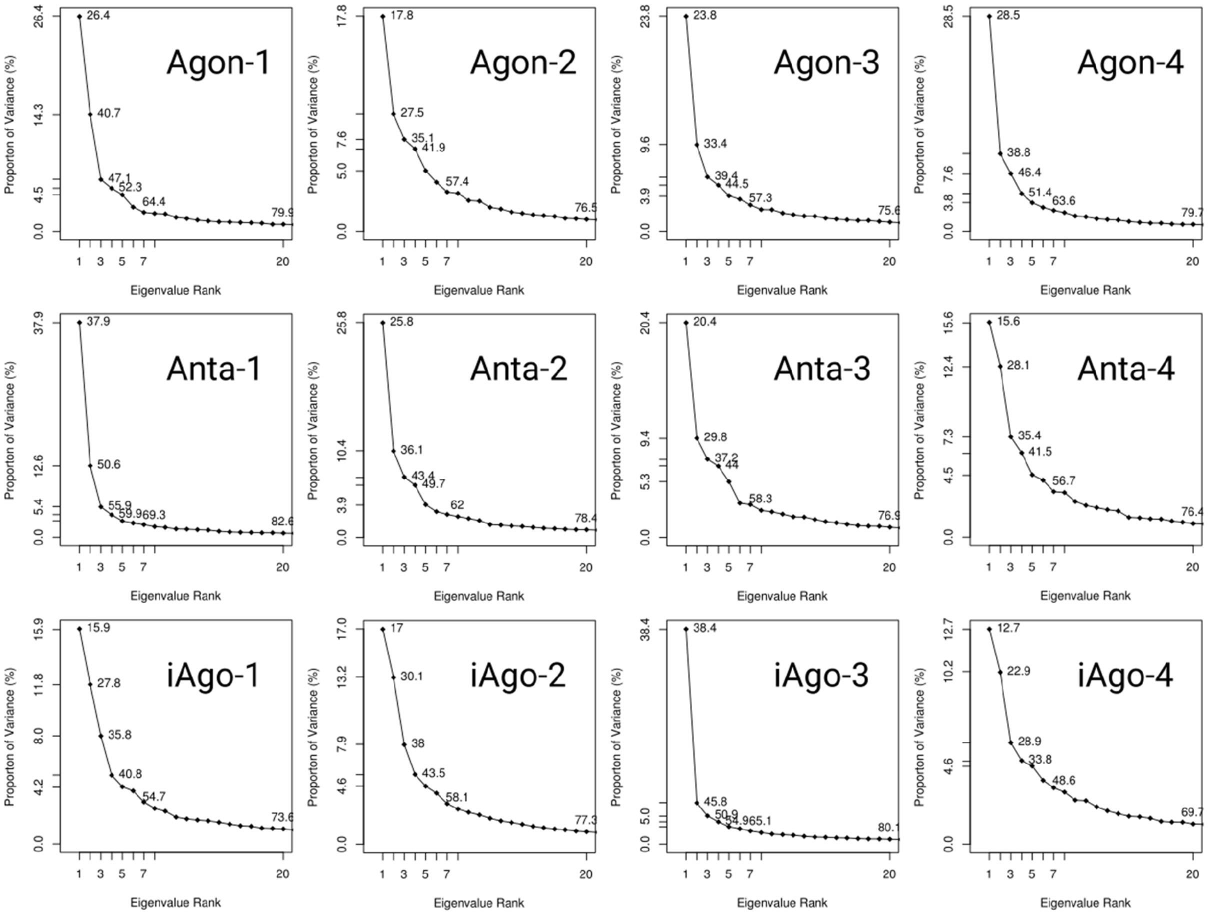

Figure Suppl. 5 | The proportion of mean square displacements (or variance %) of atoms' positional fluctuations versus their corresponding eigenvalue rank in all 12 systems.
